# Using the K‐Means Node Clustering Method and ROC Curve Analysis to Define Cut‐Off Scores for the Caregiving System Scale

**DOI:** 10.1002/ijop.70038

**Published:** 2025-03-09

**Authors:** Daiana Colledani, Mario Mikulincer, Phillip R. Shaver, Anna Maria Meneghini

**Affiliations:** ^1^ Department of Psychology, Faculty of Medicine and Psychology Sapienza University of Rome Rome Italy; ^2^ Baruch Ivcher School of Psychology Reichman University Herzliya Israel; ^3^ Department of Psychology University of California Davis Davis California USA; ^4^ Department of Human Sciences University of Verona Verona Italy

**Keywords:** caregiving profiles, CSS, cut‐off scores, K‐means node, ROC curves

## Abstract

This study was conducted to establish cut‐off scores for the subscales of the Caregiving System Scale (CSS). Two samples of Italian adults (*N*'s = 682 and 227) completed the CSS. In the first sample, K‐means node clustering and ROC curve analyses were conducted. Four caregiving profiles were identified and cut‐off scores were calculated for classifying participants into these profiles. In the second sample, participants completed the CSS and the Attachment Style Questionnaire. Findings supported the presence of unique CSS profiles and meaningful connections between them and attachment orientations. This work offers a method for determining cut‐off scores when gold‐standard measures needed to run ROC curve analyses are unavailable.

## 1 Introduction

According to attachment theory, human beings are born with a psychobiological system (the *caregiving behavioural system*) that motivates them to care for others who are in need (e.g., Bowlby 1982; George and Solomon 2008; Meneghini et al. 2018). The functioning of this system develops over the life span and is influenced by individuals' subjective experiences of care provision, resulting in mental representations of self and others in caregiving contexts (e.g., Collins et al. 2010; Mikulincer and Shaver 2015, 2016). The caregiving behavioural system works adaptively when individuals develop positive representations of self and others (Collins et al. 2010). In this case, people consider themselves effective caregivers and view others as worthy of consideration. They are sensitive and responsive to others' needs but are also able to suspend caregiving when a relationship partner does not require or request care or attention.

Conversely, when individuals experience recurrent failures in assisting others, the caregiving system develops non‐optimally, and negative mental representations of self and others ensue (Mikulincer and Shaver 2016).

Maladaptive caregiving strategies can take the form of either anxious hyperactivation (HY) or avoidant deactivation (DE) of the caregiving system (George and Solomon 2008; Shaver et al. 2010). Over‐activated caregivers tend to exaggerate the needs of others and are always eager to help, but often do so through intrusive, anxious and poorly timed caregiving actions. These behaviours may be motivated by a desire to feel appreciated, competent, indispensable and effective as a caregiver. Conversely, deactivated caregivers tend to withdraw cognitively, emotionally and behaviourally from people who seek care. Deactivated caregivers demonstrate a lack of responsiveness and empathy for others and reduced attention to their needs. They tend to withdraw from caregiving by avoiding situations in which another person may require care and support.

From an assessment perspective, the combination of these HY and DE strategies suggests specific functioning profiles of the caregiving system. Low levels of both strategies indicate functional/optimal caregiving activation (FU); high levels of HY and low levels of DE denote an anxious/intrusive type of caregiving (AX); high levels of DE and low levels of HY characterise an inhibited or avoidant type of caregiving (AV) and high levels of both strategies indicate a conflicted, especially problematic type of caregiving (PR; e.g., Shaver et al. 2010). These caregiving tendencies may be relevant for understanding a person's cognitions, feelings and behaviour in caregiving contexts and may be important clinically.

According to Mikulincer and Shaver (2016), these caregiving profiles are associated with a person's attachment orientations—systematic patterns of relational expectations, emotions and behaviours that result from a particular history of interactions with close relationship partners (Shaver and Fraley 2000). Avoidant attachment, which is characterised by repeated efforts to maintain independence (both emotional and behavioural) from others and by a lack of trust in them, tends to contribute to caregiving‐system deactivation. People with an avoidant attachment pattern often express critical and cynical opinions about others and feel discomfort with relational closeness, especially if partners feel vulnerable or express needs for care and attention (e.g., Colledani et al. 2022; Kunce and Shaver 1994). In contrast, anxious attachment, typical of individuals who desire closeness but fear rejection, seems to favour HY of the caregiving system, guided by a desire to be loved and valued by others rather than by a prosocial motive to care for others (Kunce and Shaver 1994). People who feel secure with regard to attachment do not feel uncomfortable about providing care to needy others, and their caregiving efforts are generally guided by altruistic motives (Mikulincer and Shaver 2016).

Shaver et al. (2010) developed a questionnaire to measure individual differences in the two defensive caregiving strategies (i.e., HY and DE), the Caregiving System Scale (CSS). The 20‐item CSS includes two subscales, one each tapping HY and DE tendencies (10 items per subscale). The two subscales assess orthogonal dimensions that form a two‐dimensional space in which different caregiving orientations can be represented (Meneghini et al. 2015). Previous studies conducted in Israel, the United States, Italy and Portugal supported the adequacy of the scale's psychometric properties (e.g., Meneghini et al. 2015; Shaver et al. 2010). These studies also provided support for the construct validity of the scale. The DE subscale was found to be negatively associated with empathy and compassion, involvement in volunteering activities and endorsement of prosocial values (e.g., Meneghini et al. 2015, 2018; Shaver et al. 2010). The HY subscale was associated with lower feelings of self‐efficacy as a caregiver and higher distress in the presence of needy others (Shaver et al. 2010). Interestingly, Shaver et al. (2010) also found that both CSS subscales were negatively associated with the ability to provide care and support within parent–child and couple relationships.

The CSS has been used to examine associations between these maladaptive strategies and other theoretically related constructs (e.g., Colledani et al. 2022; Meneghini et al. 2015, 2018; Moreira et al. 2018; Shaver et al. 2010; Tricarico et al. 2024). However, none of these studies have provided cut‐off scores to differentiate individuals with high and low scores on the two subscales. Consequently, although the scale is useful for the scientific study of associations between deactivating and hyperactivating strategies, on one hand and other relevant constructs, its use in applied settings can be limited. In fact, the lack of cut‐off scores makes it difficult for mental health professionals to differentiate individuals with maladaptive caregiving orientations from those with adaptive patterns, thus limiting the ability to identify caregivers who might endanger their partner's mental health and functioning in various kinds of relationships (e.g., romantic relationships, see Reizer et al. 2014, or nurse–patient relationships). Cut‐off scores are also valuable markers for providing personalised support to caregivers who might need counselling and assistance in mitigating caregiving‐related anxious or avoidant tendencies. Early identification and appropriate interventions are essential for mitigating the negative effects of maladaptive orientations for both caregivers and care recipients (Joo et al. 2022; King et al. 2012).

The most familiar method for defining cut‐off scores on psychological scales is the receiver operating characteristic (ROC) curve. To apply this method, two kinds of data are required: the scale scores of a group of respondents and an independent gold standard measure indicating their actual location on the measured characteristic (e.g., diagnosed vs. non‐diagnosed) regardless of their scale scores (Carter et al. 2016). In psychology, gold‐standard measures are usually provided by experienced professionals through clinical observations based on well‐defined criteria (e.g., DSM criteria) or obtained through the application of valid diagnostic tests. Similarly, in the clinical field, gold‐standard measures can be represented by discrete diagnostic criteria or proxies (e.g., gambling frequency as a proxy of problem gambling; insomnia as a proxy of depression) that exhibit at least a moderate association with the variable of interest. However, such criteria and assessments are not always readily available or easily identifiable. For example, for the CSS, there are no established criteria for identifying people with high or low levels of caregiving DE or HY. Additionally, potential proxies may be too generic. This makes the application of ROC curve analysis and the definition of effective cut‐off scores challenging. In this context, it is beneficial to consider alternative methods for identifying cut‐off scores that do not rely solely on traditional gold‐standard or proxy measures that may turn out to be unreliable.

In the present study, we identify cut‐off scores for the two CSS dimensions (DE, HY) using a two‐step procedure that does not require external gold‐standard evaluations. In the first step, we used the K‐means node clustering method and ROC curve analyses to establish cut‐off scores for classifying high and low scorers on each CSS subscale. The K‐means method allows for the grouping of individual data points into distinct clusters without requiring a specific target variable or criterion. In the present study, we employed this clustering method to uncover four distinct clusters (i.e., FU, AX, AV and PR patterns of caregiving) based on participants' DE and HY scores. ROC curves were then used to determine the optimal cut‐off scores for DE and HY with which to accurately predict participants' locations in particular clusters.

In the second step, the cut‐off scores determined in the first step were used to categorise a new sample of respondents into the four caregiving patterns (FU, AX, AV, PR) based on their scores on the CSS DE and HY subscales. To check the stability of the proposed solution, we compared the clustering achieved from using the first‐step cut‐off scores with that obtained by re‐running the K‐means method on the second sample. We also verified the appropriateness of the clustering achieved from using the first‐step cut‐off scores by examining mean score differences in attachment orientations.

Based on attachment theory and research (Shaver and Mikulincer 2010; Shaver et al. 2019), we hypothesised that, if the clustering process was effective, participants in the FU group (low DE and HY) would report higher levels of attachment security. Moreover, participants in the AX and PR groups, who score higher than the cut‐off score on the CSS HY scale, would report higher levels of attachment anxiety than participants in the other two groups (FU, AV). In addition, participants in the AV and PR groups, who score higher than the cut‐off score on the CSS DE scale, would report higher levels of avoidant attachment than participants in the other two groups (FU, AX).

## 2 Materials and Methods

### 2.1 Participants and Procedure

Two groups of participants took part in the study. The total sample includes 909 individuals (female 70.3%; mean age = 26.98, SD = 11.58; one respondent did not indicate his/her gender) who were enrolled from the general population (aged between 18 and 73 years) in different Italian regions. The questionnaire was distributed via email, following a snowball procedure (using a mailing list). No specific inclusion/exclusion criteria were defined, except that participants had to be native Italian speakers and at least 18 years old. To access the questionnaire, participants were asked to accept an electronic informed consent statement detailing the study's aim, task duration and the voluntary, anonymous nature of their participation (only gender and age were requested). Participants received no compensation. The research project was approved by the local Ethics Committee for Psychological Research.

The first group of respondents included 682 individuals (female 72.3%; mean age = 28.56, SD = 12.72) who completed the 20 items of the CSS. The second group included 227 participants (female 64.3%; mean age = 22.25, SD = 4.52) who completed the CSS and another measure that assessed attachment orientations.

### 2.2 Measures

All participants were presented with the Italian version of the 20‐item CSS (Meneghini et al. 2015). This scale comprised two 10‐item subscales: the caregiving DE subscale (e.g., ‘I don't often feel an urge to help others’) and the caregiving HY subscale (e.g., ‘I often get anxious when I think nobody needs my help’). Participants were asked to focus on the way they usually behave and feel when they are helping other people and to rate the extent to which each item is representative of their feelings and behaviour. Ratings were made on a 7‐point scale ranging from 1 (*not at all*) to 7 (*very much*). On each subscale, higher scores indicate higher levels of caregiving DE or HY. In the current samples (combined *N* = 909), Cronbach's *α* coefficients were quite satisfactory (*α* = 0.83 and 0.84 for DE and HY, respectively).

A second sample of 227 individuals answered the CSS together with the Italian version of the Attachment Style Questionnaire (ASQ; Fossati et al. 2003). The ASQ is a 40‐item self‐report scale assessing three adult attachment dimensions: attachment security (8 items), attachment‐related avoidance (17 items, discomfort with closeness and appraising relationships as personally irrelevant) and attachment anxiety (15 items; need for approval and fear of being rejected and abandoned). Participants rated their agreement with each item on a 6‐point scale ranging from 1 (*totally disagree*) to 6 (*totally agree*). In the current sample, Cronbach's *α*s for anxious, avoidant and secure attachment subscales were 0.85, 0.79 and 0.70, respectively. On this basis, we computed three total scores by averaging items in each subscale, with higher scores indicating higher levels of security, anxiety or avoidance.

### 2.3 Statistical Analyses

To identify cut‐off scores for the DE and HY subscales of the CSS, the K‐Means node clustering method and ROC curve analyses were applied to the data of the first sample (*N* = 682). The K‐Means node is a clustering method for grouping individual data points into distinct groups without using a specific target or criterion variable (Lorr 1983). This method of clustering is called *unsupervised learning* because, rather than trying to predict an outcome, it attempts to identify (learn) a pattern within a set of input data. The K‐Means node aims to uncover groups (or clusters) of records that are as similar as possible within them and as different as possible between them. Once the number of desired clusters is specified, the K‐Means method selects some data points to be used as starting points (i.e., seeds) for clustering. Next, the algorithm begins an iterative process in which all records are assigned to the cluster to which they are most similar (minimising the within‐group sum of squared error as measured by Euclidean distances). After all cases have been assigned to a specific cluster, the algorithm calculates new cluster centroids (considering the records assigned to each cluster) and reassigns records to the appropriate cluster according to the new centroids. The process continues until clusters become stable (i.e., the change between iterations does not exceed a defined threshold). This approach makes it possible to identify distinct groups within the data.

In the present study, the K‐Means node method was applied to the standardised sum scores of the DE and HY subscales. Because outliers and the missing data pattern can bias the results of the K‐Means node method (Wang et al. 2019; Lopes and Gosling 2020), the data set was checked to detect a missing data pattern and multivariate outliers. Outliers were checked using the Mahalanobis distance method (for DE and HY).

As suggested in the literature (Witten et al. 2016), we set the procedure for four clusters based on theoretical expectations (i.e., the FU, AX, AV and PR patterns of caregiving; e.g., Shaver et al. 2010). In addition, the Krzanowski–Lai (KL) index (Krzanowski and Lai 1988) was used to evaluate the quality of the four‐cluster solution compared to other solutions with three and five clusters, respectively. This coefficient, which is also known as the average silhouette width (ASW) or KL index, is a measure of cohesion and separation and indicates how much a data point is close (similar) to the other records of its own cluster (cohesion) compared to those of the other clusters (separation). Larger values of the KL index indicate better‐defined clusters with good separation and cohesion.

The optimal number of clusters can be determined by identifying the solution (number of cluster) in which the KL index is the largest (Krzanowski and Lai 1988). The K‐means algorithm is sensitive to the choice of the initial seed (Khan 2012). To address this issue and obtain a stable solution, the algorithm was run 1000 times with different seeds. The solution that appeared most frequently was selected to represent the data.

After having verified the adequacy of the clustering solution, the resulting clusters were examined. We expected to identify four specific clusters: participants with high scores on both CSS subscales (HH, the PR caregiving profile), participants with low scores on both CSS subscales (LL, the FU caregiving profile), participants with high DE scores and low HY scores (HL, the AV caregiving profile) and participants with low DE scores and high HY scores (LH, the AX caregiving profile). Based on this analysis, we defined two dichotomous variables distinguishing participants with high and low levels of DE and HY. The first variable was created by coding participants in the HH and HL clusters as ‘high DE’ (1) and those in the LL and LH clusters as ‘low DE’ (0). Analogously, the second dichotomous variable was created by coding participants in the HH and LH clusters as ‘high HY’ (1) and those in the LL and HL clusters as ‘low HY’ (0).

The two dichotomous variables created using this method were used as gold‐standard measures to run ROC curve analyses (Carter et al. 2016). Given a gold standard criterion (i.e., classification variable), ROC curves aim to identify the score, among all the possible test scores, that allows for correctly classifying the largest number of participants according to the levels of the gold‐standard variable. The sensitivities (i.e., proportions of cases correctly classified as having the considered characteristic or ‘true positive rates’) and specificities (i.e., proportions of cases correctly classified as not having the considered characteristic or ‘true negative rates’) of all possible scores are tabulated and the score maximising both sensitivity and specificity is selected as the best cut‐off score (the cut‐off score that most accurately categorises participants based on the levels of the dichotomous variables). In this work, ROC curves were run on the sum scores of the DE and HY subscales of the CSS to identify the cut‐off scores that better differentiated participants falling into the levels of the gold‐standard measures (i.e., the dichotomous variables differentiating between high and low deactivated caregivers and between high and low hyperactivated caregivers).

The optimal cut‐off scores for both subscales were determined by evaluating three criteria: the Youden index (J; Youden 1950), the angle of the point closest to (0, 1) in the ROC plane approach (ER; Perkins and Schisterman 2006) and the Liu index (CZ; Liu 2012). The Youden index identifies the optimal cut‐off score as the score that maximises the difference between the true positive rate (i.e., sensitivity) and the false positive rate (i.e., 1 − specificity) over all possible cut‐off scores (i.e., sensitivity + specificity − 1). The ROC plane approach defines the optimal cut‐off score as the point that minimises the Euclidean distance between the ROC curve and the point (0, 1) (ER= $\sqrt{{\left(1-\text{specificity}\right)}^{2}+{\left(\text{sensitvity}-1\right)}^{2}}$). The Liu index identifies the optimal cut‐off point as the one that maximises the product of sensitivity and specificity (sensitivity × specificity).

The cut‐off scores identified with these methods were subsequently applied to the data of the second sample (*N* = 227). In this step, respondents were classified as ‘high‐ or low‐DE’ and ‘high‐ or low‐HY’ based on the cut‐off scores identified in the first sample. Based on these classifications, participants were then assigned to one of four caregiving profiles (FU, AX, AV, PR). Finally, to validate the clustering of participants according to their scores on the DE and HY subscales of the CSS, three analyses of variance (ANOVAs) were run examining differences between the four caregiving profiles (FU, AX, AV, PR) in terms of attachment orientations (secure, anxious, and avoidant attachment).

## 3 Results

**FIGURE 1:**
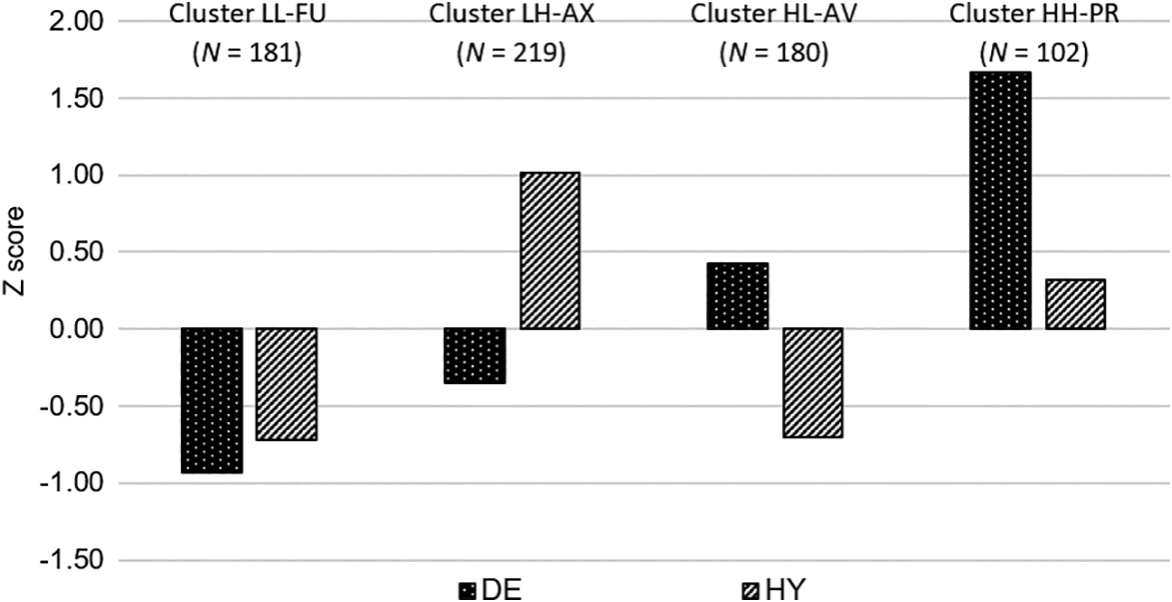
Standardised scores on the DE and HY scales for the four clusters resulting from the K‐means method (*N* = 682). AV = avoidant caregiving (high DE and low HY, HL); AX = anxious caregiving (low DE and high HY, LH); FU = functional caregiving (low DE and HY, LL); PR = problematic caregiving (high DE and HY, HH); DE = deactivation; HY = hyperactivation.

First, descriptive statistics (*N* = 682; DE: *M* = 21.61, SD = 8.85, Skewness = 0.70, Kurtosis = 0.14; HY: *M* = 32.95, SD = 10.58, Skewness = 0.23, Kurtosis = −0.32; correlation between DE and HY was 0.03, *p* = 0.39) and reliability coefficients (αs 0.80 and 0.83 for DE and HY, respectively) were computed for the two CSS subscales (no missing data patterns were found in the CSS item responses, and no multivariate outliers were observed). Next, the K‐means analysis was run on standardised scores of the DE and HY subscales asking for four clusters (Figure 1). The KL index values for the three‐, four‐ and five‐cluster solutions were 1.12, 9.44 and 0.53, respectively. These results suggested that the four‐cluster solution was the most suitable for the data. The algorithm was run 1000 times with different seed values to determine four clusters. This analysis identified a recurring cluster structure that appeared 308 times, and this pattern was selected to represent the data. In this clustering solution, the pattern of DE and HY scores of the four clusters were in line with expectations and clearly represented the four caregiving profiles: FU (relatively low scores on the two CSS subscales, *N* = 181), AX (relatively low DE scores and high HY scores, *N* = 219), AV (relatively high DE scores and low HY scores, *N* = 180) and PR (relatively high scores on the two CSS subscales, *N* = 102).

Based on the results of the K‐means analysis, we created two dichotomous variables. The first differentiated participants scoring high on the DE subscale (participants in the AV and PR clusters; coded as 1; *N* = 282, 41.3%) from those scoring low on this subscale (i.e., participants in the FU and AX clusters; coded as 0; *N* = 400, 58.7%). The second variable distinguished participants scoring high on the HY subscale (participants in the AX and PR clusters; coded as 1; *N* = 321, 47.1%) from those scoring low on this subscale (participants in the FU and AV clusters; coded as 0; *N* = 361, 52.9%).

**FIGURE 2:**
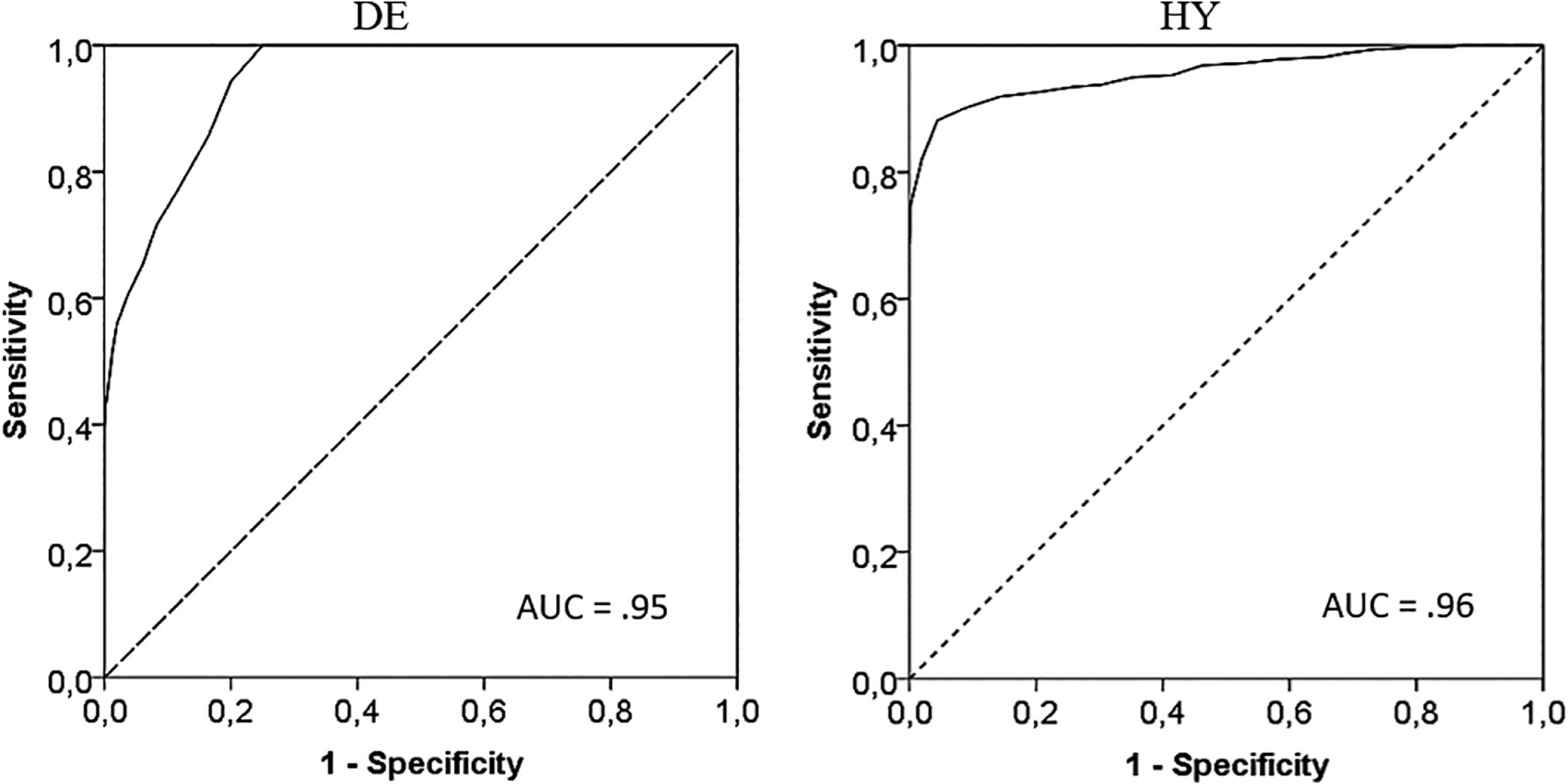
ROC curve. Receiver operating characteristic (ROC) curves for the DE and HY subscales of the CSS (*N* = 682). AUC = area under the curve.

These two dichotomous variables were used to run ROC‐curve analyses on the sum scores of the DE and HY subscales (Figure 2). For the DE subscale, the Youden index indicated a score ≥ 20 as the optimal cut‐off score to differentiate participants with high DE scores from those with low DE scores (sensitivity = 1.00, specificity = 0.75; *J* = 0.75). However, both the ER and the CZ indices indicated a score of ≥ 21 as the optimal cut‐off (sensitivity = 0.94, specificity = 0.80; ER = 0.76; CZ = 0.21). Based on the results of the three criteria, a score of ≥ 21 was selected as the optimal cut‐off score. With regard to the HY scale, all three indices unanimously suggested a cut‐off score of ≥ 35 as the optimal cut‐off score to differentiate between participants with high and low scale scores (sensitivity = 0.88, specificity = 0.96; *J* = 1.84; ER = 0.84; CZ = 0.13).

**FIGURE 3:**
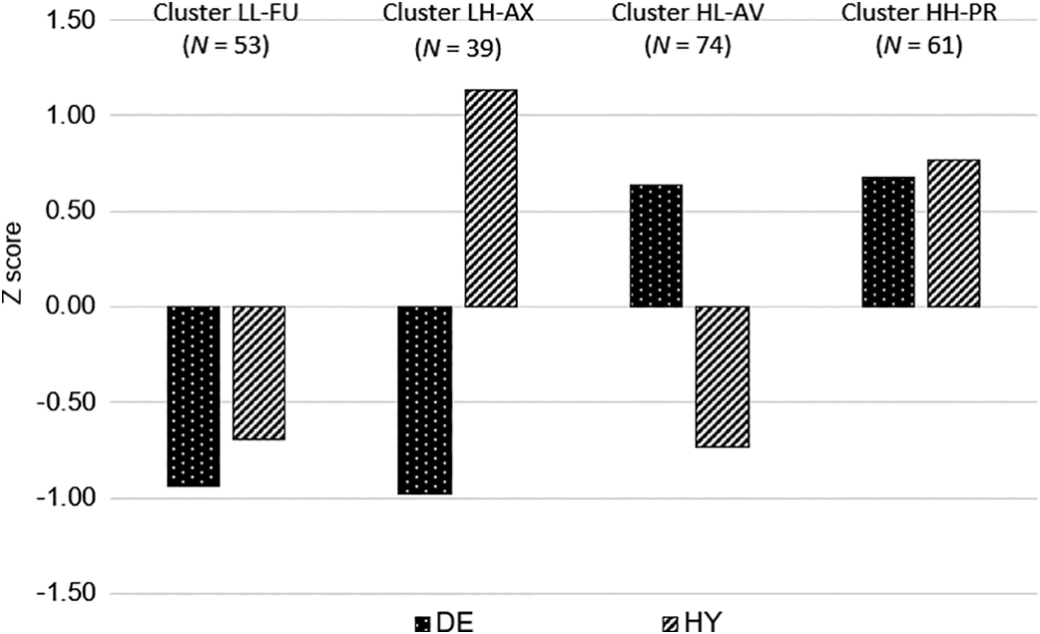
The standardised mean scores on the DE and HY scales for the four caregiving patterns defined using the cut‐off scores (*N* = 227). The four groups were defined using the cut‐off scores (i.e., ≥ 21 and ≥ 35, for DE and HY, respectively) identified in the first sample (*N* = 227). AV = avoidant caregiving (high DE and low HY, HL); AX = anxious caregiving (low DE and high HY, LH); FU = functional caregiving (low DE and HY; LL); PR = problematic caregiving (high DE and HY, HH); DE = deactivation; HY = hyperactivation.

To cross‐validate the cut‐off scores identified in the first data set (*N* = 682), these scores were applied to the second data set (*N* = 227). Specifically, the two cut‐off scores (≥ 21 for DE and ≥ 35 for HY) were used to classify participants based on their sum scores on the DE and HY subscales. This resulted in the following categorizations: 135 participants were coded as ‘high DE’ (score ≥ 21), 92 as ‘low DE’ (score < 21), 100 as ‘high‐HY’ (score ≥ 35) and 127 as ‘low‐HY’ (score < 35). Concerning the four caregiving profiles, 53 participants were sorted into the FU group, 74 in the AX group, 39 in the AV group and 61 in the PR group. As shown in Figure 3, the emerging profiles closely resemble those obtained with the K‐node method in the first sample, which were in line with theoretical expectations.

To investigate the suitability of the clustering resulting from the use of the first‐sample cut‐off scores (≥ 21 and ≥ 35), we conducted ANOVAs testing differences in attachment orientations between the four caregiving pattern groups (see relevant means, SDs and *F*‐ratios in Table 1). The findings were in line with predictions (see Table 1). Participants in the FU group scored higher on the secure attachment subscale than participants in the other three groups (significantly higher than PR and AX). Furthermore, participants in the AX and PR groups scored higher on the anxious attachment subscale than participants in the FU and AV groups, and participants in the AV and PR groups scored higher on the avoidant attachment subscale than participants in the FU and AX groups (all pairwise comparisons between group means were significant, *p* < 0.05; excluding the comparison between AX and AV on anxious and avoidant attachment; see Table 1).^1^

**TABLE 1:** Means, SDs and F‐ratios for attachment orientations as a function of the four caregiving patterns.

Variable		LL‐FU (*N* = 53)	HL‐AV (*N* = 74)	LH‐AX (*N* = 39)	HH‐PR (*N* = 61)	*F*	*η* ^2^
Secure attachment	*M*	4.19 a, b	3.98 c	3.85 a, d	3.47 b, c, d	14.88***	0.17
	SD	0.50	0.61	0.69	0.63		
Anxious attachment	*M*	2.92 a, b	3.09 c	3.44 a, d	3.91 b, c, d	22.16***	0.23
	SD	0.61	0.75	0.74	0.75		
Avoidant attachment	*M*	2.84 a, b	3.28 a	3.08 c	3.48 b, c	13.88***	0.16
	SD	0.55	0.55	0.61	0.50		

*Note:* Means with the same letters within a row were significantly different at *α* 0.05.

Abbreviations: AX = anxious caregiving; AV = avoidant caregiving; FU = functional caregiving; PR = problematic caregiving.

*** *p* < 0.001.

## 4 Discussion

Caregiving orientations are conceptualised as relatively stable patterns of responses to others' needs (Mikulincer and Shaver 2016). Whereas caregiving HY includes coercive, intrusive and tactless caregiving behaviours, caregiving DE involves emotional detachment from situations that demand care provision. An instrument, the CSS, was developed to assess these orientations. Although the CSS has been shown to be an effective tool for assessing caregiving DE and HY in different cultural contexts (Meneghini et al. 2015; Moreira et al. 2018; Shaver et al. 2010), previous studies have not provided valid cut‐off scores to differentiate between high and low scores on each caregiving dimension. This makes it difficult to classify individuals into one of the four caregiving patterns, as defined by scores on the two CSS dimensions, and then to provide personalised counselling and assistance when AX, AV or PR patterns of caregiving become dominant when a person is called upon to provide care to a needy other.

Cut‐offs provide information that can be used by researchers and practitioners who need to identify when HY and DE scores suggest suboptimal caregiving behaviours. For example, they could be valuable in clinical practice to train and support professional caregivers who may experience difficulties during their caregiving activities. In addition, nowadays, informal caregivers represent a large proportion of the population (Tur‐Sinai et al. 2020). Informal caregivers are people who, throughout their lives, find themselves in the position of having to assume the role of caregiver, as it becomes necessary for them to provide care for a relative (or a friend) in permanent need of special support due to chronic illness, normal or pathological ageing or disability (as in the case of parenting children with neuroatypical development). Classifying them into one of the four caregiving patterns, as defined by scores on the two CSS dimensions, can help in planning targeted supportive interventions for those who experience caregiving fatigue and emotional burnout.

Identifying maladaptive caregiving orientations and implementing appropriate interventions are extremely important for promoting better outcomes for both caregivers and care recipients (Joo et al. 2022; King et al. 2012). In addition, as Reizer et al. (2014) have shown, non‐optimal caregiving activations can impair relationship satisfaction in romantic relationships. Therefore, knowing about partners' caregiving profiles may help couple therapists repair relational wounds and restore relationship quality.

In the present study, the K‐means clustering method and ROC‐curve analyses were run to identify the optimal cut‐off scores to differentiate individuals scoring high and low on the two subscales of the CSS. This procedure allowed the identification of optimal cut‐off scores for the DE subscale (i.e., ≥ 21) and the HY subscale (i.e., ≥ 35) of the CSS. Interestingly, the results of the K‐means clustering method fit perfectly with theoretical expectations. The four emerging groups clearly reflected the four expected patterns of caregiving: The FU pattern defined by low scores on DE and HY, the AX pattern defined by high HY and low DE scores, the AV pattern defined by low HY and high DE scores, and the PR pattern defined by high scores on the two CSS subscales. Also, ROC curve analyses defined two highly accurate cut‐off scores (AUC 0.95 and 0.96).

The results were cross‐validated in a second independent sample by examining the differences in attachment orientation between the four groups of caregiving patterns identified using the cut‐off scores defined in the first sample. The differences in mean scores were perfectly in line with expectations and showed that participants in the FU group scored highest on secure attachment, those in the AX and PR groups scored higher on anxious attachment, and those in the AV and PR groups scored higher on avoidant attachment. These findings support the construct validity of participants' classification into four caregiving patterns.

Although the K‐means clustering method appeared appropriate for our analysis, it is crucial to recognise its inherent limitations. Research has shown that this method can exhibit bias, particularly when applied to data sets characterised by skewed distributions, small sample sizes or highly correlated variables. Empirical evidence suggests that even minor violations of these assumptions can lead clustering methods to inaccurately determine the appropriate number of distinct groups (Toffalini et al. 2022, 2024). For example, highly correlated variables may considerably inflate the number of identified clusters, resulting in a notable increase in false positives (Type I error; Toffalini et al. 2022, 2024). Moreover, the K‐means clustering method is sensitive to outliers and initial conditions (Khan 2012; Lopes and Gosling 2020; Wang et al. 2019). In the present study, the K‐means clustering method was applied in a reasonably robust context, characterised by a sufficiently large sample size, low correlations among the indicators (consistent with the literature; Meneghini et al. 2015; Moreira et al. 2018), the absence of outliers or missing data, and no violations of distributional assumptions. Furthermore, considering the sensitivity of the K‐means algorithm to initial seeds (Khan 2012), the algorithm was executed 1000 times with different seeds to ensure a stable solution. These factors contribute to the soundness of the approach taken in the present study. However, as outlined in the relevant literature (Toffalini et al. 2022, 2024), it is advisable to thoroughly evaluate the aforementioned aspects before implementing the method and, where feasible, to quantify potential inferential risks a priori.

The clustering of participants into one of four caregiving patterns can contribute to understanding how a person's actions and thoughts tend to be organised when he or she is called upon to provide help and support to others in distress. Each pattern indicates the prevailing way in which the individual tends to respond. However, it should be kept in mind that actual responses are also influenced by features of the situation, current motives, intentions, the affective state of the helper and the identity of the needy person, and that the same person might enact different caregiving responses in different situations (Mikulincer and Shaver 2016).

Before ending this discussion, we should mention two methodological limitations of this study: its exclusive reliance on self‐report measures and its cross‐sectional design. Future studies should consider the use of alternative behavioural measures and prospective longitudinal designs. In addition, future studies should aim to collect data from individuals who are actively caring for someone in need (e.g., professional caregivers or those caring for a sick spouse or child) and replicate our findings using diverse samples from different countries, cultures and religions.

The present study employs a well‐established methodology and precise theoretical foundations (e.g., Shaver et al. 2010), making the approach methodologically and theoretically grounded. However, future studies could consider extending the current findings to other caregiving‐related scales. Additionally, they could explore the feasibility of utilising Latent Profile Analysis (LPA) instead of the K‐means method (see Dal Corso et al. 2020; Paschke et al. 2021). Finally, future research could examine the stability of caregiving profiles over time and the effectiveness of interventions aimed at promoting FU/optimal caregiving while addressing AX, AV or PR caregiving patterns. These endeavours would not only deepen our understanding of caregiving orientations but also contribute to the well‐being and relationship quality of both caregivers and care recipients.

## Ethics Statement

All procedures performed in studies involving human participants were in accordance with the ethical standards of the institutional research committee at University of Padua [protocol approval code: 5B755BA79AD5DEEC3FFA19DED2E0DC19] and with the 1964 Helsinki Declaration and its later amendments or comparable ethical standards.

## Consent

Informed consent was obtained from all individual adult participants included in the study.

## Conflicts of Interest

The authors declare no conflicts of interest.

## Data Availability

The data used in this work are available upon request from the corresponding author. R scripts used to conduct K‐means clustering are available in a public repository (https://osf.io/64vm5/?view_only=bf1af751de7e43819efbee899a22b280).

## Endnotes

^1^
For all ANOVAs, post hoc power analyses were performed using G*Power 3.1.9.7. The results showed that with a total sample size of *N* = 227, divided into four groups, a significance level (*α*) of 0.05, and medium to large effect sizes (*f* ranging from 0.38 to 0.47), a statistical power (1 − *β*) of 0.99 was achieved.
